# Effects of community action on animal vaccination uptake, antimicrobial usage, and farmers’ wellbeing in Ghana: study protocol for a cluster-randomized controlled trial

**DOI:** 10.1016/j.onehlt.2024.100952

**Published:** 2024-12-15

**Authors:** Francis Sena Nuvey, Günther Fink, Jan Hattendorf, Daniel T. Haydon, Gilbert Fokou, Kennedy Kwasi Addo, Jakob Zinsstag, Clemence Esse-Dibby, Bassirou Bonfoh

**Affiliations:** a Friedrich-Loeffler-Institut, Südufer 10, 17493 Greifswald, Germany; b Swiss Tropical and Public Health Institute, Kreuzstrasse 2, 4123 Allschwil, Switzerland; c Centre Suisse de Recherches Scientifiques en Côte d’Ivoire, Abidjan BP 1303, Côte d’Ivoire; d School of Biodiversity, One Health and Veterinary Medicine, College of Medical, Veterinary and Life Sciences, University of Glasgow, G12 8QQ Glasgow, UK; e Department of Bacteriology, Noguchi Memorial Institute for Medical Research, University of Ghana, Accra, P.O. Box LG 581, Ghana

**Keywords:** One Health, Infectious livestock diseases, Vaccination, Antimicrobial use, Wellbeing, Transdisciplinary

## Abstract

Infectious animal diseases represent a major constraint to livestock productivity, food security and wellbeing in many developing countries. To mitigate these impacts, farmers frequently use antimicrobials without professional advice, potentially yielding drug residues in livestock products and the food chain, as well as resistant antimicrobial genes. Recent studies identified Contagious Bovine Pleuropneumonia (CBPP) and Peste des Petits Ruminants (PPR) as the diseases most negatively affecting ruminant livestock productivity and farmers’ wellbeing in Ghana. Despite the approval and availability of effective CBPP and PPR vaccines in Ghana, acceptability, affordability, accessibility, and availability of vaccination limit their uptake, with only 15% of farmers regularly vaccinating their herds. During formative qualitative research to identify barriers and potential intervention options, farmers suggested that establishing localized farmer intervention platforms could improve vaccine access. The main idea is the platforms enabling information exchange on livestock vaccines, enhancing service scheduling, and sharing vaccination costs among farmers living in the same locality. We now wish to test formally this hypothesis.

Through a cluster-randomized controlled trial, we aim to determine the effect of localized farmer platforms on animal vaccination uptake (primary outcome), antimicrobials use in livestock production, disease-induced mortality in livestock, and livestock farmers’ wellbeing (secondary outcomes). The intervention will be randomized at the community level. The study will involve 460 farming households across 46 rural communities (study clusters). Clusters will be randomized with equal probability to treatment and control (23 communities each). Approximately 10 households per community will be sampled for data collection at baseline, and at 6 and 12 months post-intervention, following prevailing vaccination schedules. We will conduct an intention-to-treat analysis using the available case population. The findings will inform strategies to tackle the impact of infectious livestock diseases on food security, public health and farmers’ wellbeing.

**Trial registry**: https://pactr.samrc.ac.za/; ID No.: PACTR202405854213937.

## 1 Introduction

In low-and-middle income countries (LMICs), animal diseases are highly prevalent and poorly controlled due to low performance of veterinary services and limited farmer engagement, resulting in significant herd mortalities [1]. On average, about 7% of adult cattle, 21% of calves, 15% of adult sheep and goats, and 23% of lambs and kids die from animal diseases annually in the least developed countries [2]. In LMICs, majority of households are smallholders earning less than USD 60 monthly in agricultural income [3]. In contrast, the average market value of ruminant livestock in Africa ranges between USD 120 for one sheep or goat and USD 600 for one cattle [4]. The livestock losses negatively affects productivity of the livestock sector, livelihoods of farming households and contributes to food insecurity, particularly for vulnerable households in LMICs [5]. Disease-related mortalities also adversely affect farmers’ wellbeing, particularly on the psychological, physical, and social domains [[6], [7], [8]].

What is more, veterinary services delivery has been ineffective in many LMICs in practice, due to inadequate public investment in the veterinary sector, with significant shortfalls in human and material resources [9]. This lack of investments is often attributed to the implementation of drastic structural adjustment policies during the 1980s aimed at addressing high indebtedness levels of LMICs [10]. In addition, veterinary medicines are poorly regulated. A recent review showed that 80% of African countries lack capacity to control the registration, import and production, distribution and usage of veterinary medicines. There is also a lack of drug residue testing programmes in more than two-thirds of the countries [11]. Thus, an overwhelming majority of farms use antimicrobials regularly, without professional advice [12,13].

The high usage of antimicrobials coupled with a lack of residue testing programmes and inadequate regulatory capacity within the food chain, could foster the persistence of drug residues in livestock products, especially when treated livestock are slaughtered prior to recommended withdrawal periods, and accelerate the development of related antimicrobial resistant pathogens [14,15]. Given the apparent shortfalls in veterinary resources, a reduction in occurrence of diseases, particularly through disease prevention strategies, would greatly reduce the disease burden, and workload on veterinary personnel.

Effective control of infectious diseases is mainly achieved with rapid diagnostic tools and effective vaccination strategies [16,17]. However, neither strategy is currently used adequately in practice in many LMICs [11,18]. Studies in Ghana have shown that although livestock farmers and veterinary personnel identify CBPP and PPR as priority diseases affecting herds, only 15% of herds are vaccinated regularly against either disease. In addition, treatments applied by most farmers are not useful for the conditions managed. Consequently, diseases are ineffectively treated, disease-related mortality rates are high, and farmers experience deteriorating wellbeing as severity of disease-related mortalities increased [7,19].

In Ghana, both demand and supply side constraints have been identified to limit vaccination utilization. Demand-side barriers include farmers’ limited awareness of the value of vaccines, and unaffordability issues, especially when farmers must cover entire cost of vaccine vials, even if they do not have enough animals to use an entire vial. The supply-related barriers are mainly due to a limited number of trained veterinary personnel in public and private practice, and the lack of adequate veterinary health infrastructure, which limit accessibility to veterinary personnel’s services when needed [20]. The World Organization for Animal Health (WOAH, founded as OIE) data show that the average veterinary workforce per animal in Africa was approximately 3530 veterinary livestock units (VLUs) in 2019 [21]. Based on the most recent Performance of Veterinary Services (PVS) Gap Analysis conducted in 2011 in Ghana, the ratio of veterinary workforce to animals is substantially lower, more than tenfold less than the African average [22]. There is therefore a need for innovative and sustainable strategies to improve vaccination uptake, for better disease control. During group discussions in formative qualitative research aimed at co-developing sustainable interventions, farmers proposed among other measures that the formation of localized community platforms could address most of the reported challenges with vaccination access [20]. We therefore developed this intervention study to evaluate formally, the effectiveness of this proposed intervention.

## 2 Research objectives, hypothesis and study outcomes

The main objective of the study is to evaluate the effect of an innovative community-led intervention on the uptake of CBPP and PPR vaccines by livestock farmers in Ghana, and evaluate the impact of vaccination uptake on antimicrobial use in livestock farming, disease-induced livestock mortalities and farmers’ wellbeing. The study will be a cluster randomized controlled trial, which will introduce the intervention communities to a new Community Action for Vaccination Initiative (CAVI) that is expected to enable farmers to coordinate their livestock vaccination efforts by leveraging the strengths of the local group.

The study hypothesis is that CAVI has an impact on uptake of animal vaccines in farming communities. We also hypothesize that CAVI will have an impact on the usage frequency and quantity of antimicrobials in livestock production, occurrence of disease-induced animal mortalities, and wellbeing of livestock farmers.

The primary study outcome is the uptake for CBPP and PPR vaccines. The secondary outcomes include frequency and quantity of antimicrobials used by farmers, incidence of livestock herd mortalities due to disease, farmers’ wellbeing on the physical, psychological, social, and environment domains, and the acceptability and feasibility to scale of CAVI to the key stakeholders including farmers and veterinary service providers.

## 3 Methods

### 3.1 Study setting

This study will be conducted in the Mion, Pru East and Kwahu Afram Plains South (KAPS) Districts, which are representative of the northern, middle and southern farming belts of Ghana. The districts lie in the Guinea Savannah, Transition and Deciduous forest Vegetation zones, which are the primary livestock production zones in Ghana [[23], [24], [25]]. The districts were selected purposively in collaboration with the regional directors of veterinary services, using a sampling frame of farming districts located within these vegetation zones. The selected districts are mainly rural and agrarian, with about one-third of the livestock holdings of households being ruminant species. Cattle, sheep, and goats are the predominant ruminant livestock species reared in these districts. Majority of livestock rearing (53%) is for income generation – the rest is directly consumed by households, or used for other socio-cultural purposes. The livestock production system is largely extensive and dominated by small-scale farmers [26].

In rural Ghana, livestock farmers typically obtain approval from community leaders such as chiefs, local assembly members, landowners, or opinion leaders, to keep and graze their animals within the community [27]. Veterinary services in these areas are predominantly provided by veterinary paraprofessionals, who hold a minimum of a diploma in animal health. These paraprofessionals operate under the supervision of district or regional veterinarians, who hold a Doctor of Veterinary Medicine (DVM) degree, and assign them specific operational areas to deliver veterinary services [22].

To ensure resource optimization and sustainability of the intervention, this project will leverage the existing veterinary service and local authority framework within the study setting. In collaboration with the veterinary paraprofessionals assigned to the operational areas within which the intervention communities are located, we will identify individuals in each community to receive the intervention (training). The training will be conducted with support from local staff of the Veterinary Services Directorate and the Department of Agriculture in the study districts.

### 3.2 Study design

**Fig. 1:**
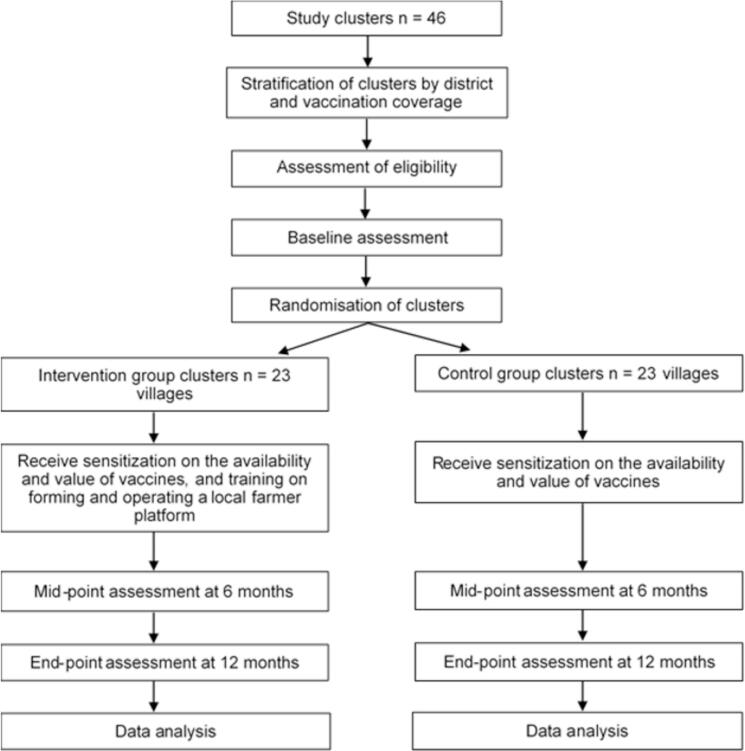
Study design

This will be a two parallel arm cluster randomized controlled trial with, wherein intervention communities will receive the CAVI package, while the control communities will receive the standard of care (individual farmers schedule vaccination with veterinary personnel on their own). The CAVI package entails training and support for selected farmers in the intervention communities to establish local platforms that facilitate scheduling vaccination activities with veterinary personnel. A group-level randomization (1:1) stratified by district was implemented in allocating the intervention to the study communities. The allocation procedures were conducted using R Software [28]. Fig. 1 below illustrates the study design.

**Fig. 2:**
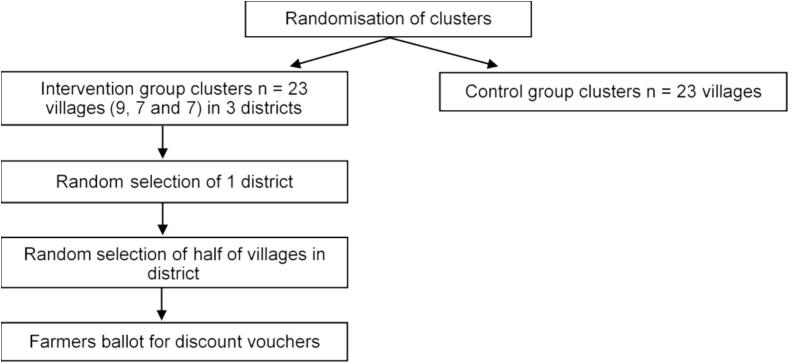
Randomization procedures in allocating the intervention

In one of the study districts, we will evaluate the effect of the provision of an add-on intervention to half of the intervention communities. This add-on intervention entails discount vouchers worth 25%, 50% and 75% of the cost of vaccinating eligible animals in a farmers’ herd. The farmers in the selected communities will ballot for the vouchers during the first local platform meeting. In the selected district, the trained individuals will additionally receive a discount voucher worth 50% of the cost of vaccinating their herd. The discount vouchers provided would be reimbursed to farmers after their payment for the vaccination services provided by the veterinary personnel within the study district. Fig. 2 below the randomization procedures in allocating the intervention.

### 3.3 Intervention (CAVI) description

The intervention package entails the training of at least two persons per community to build capacity and knowledge in understanding vaccine effectiveness, profitability, safety, and the personal and community-level benefits of vaccination. The training also covers scheduling vaccinations for priority diseases (i.e. CBPP and PPR infections) with veterinary personnel, and herd vulnerability to the diseases. The training comprises two modules designed to equip livestock farmers with the knowledge and skills necessary to establish and manage farmer cooperative platforms, to empower participants to coordinate vaccination of herds within their communities (details available in **Additional file 1**). In summary, Module 1 focuses on the benefits of collective action, emphasizing relationship-building, understanding community dynamics, and the principles of forming and managing cooperatives. Through practical exercises, group discussions, and real-life scenarios, participants will explore the importance of trust, communication, and coordination in achieving shared goals. These activities demonstrate how collective efforts can address individual challenges, improve productivity, and reduce costs associated with vaccination. Module 2 addresses the burden of the priority livestock diseases i.e., CBPP and PPR, and highlights the critical role of vaccination. Participants learn about vaccine schedules and the socio-economic benefits of improving herd health. By fostering collaboration, the intervention empowers farmers to leverage cooperative platforms for enhanced vaccine access, disease prevention, and knowledge exchange, ultimately contributing to improved livestock productivity and community resilience.

The training participants will be purposively selected in collaboration with the local community leaders and veterinary service personnel. The intervention package will be delivered after the baseline survey and reinforced at the midpoint follow-up period (6 months). Each trained individual will receive a register to keep records of local platform members, meeting days and meeting minutes. The trained individuals will each receive mobile airtime worth GHC 50 [USD 5] quarterly to facilitate their efforts in convening meetings and conducting outreach to veterinary personnel.

Since blinding is not possible, to reduce potential imbalance in dropouts between study arms and non-blinding bias, each participating farmer in the surveys in both study arms would also receive a total of GHC 50 mobile airtime over the three rounds of the survey (baseline, mid-point and end-point follow-ups) as an attention control intervention. The trained individuals will be supported to establish their respective local platform of farmers within the intervention communities, and schedule vaccination visits with veterinary personnel. The veterinary personnel will be provided with registers to record all vaccination requests received from farmers, the number of requests honored (in both study arms), and any adverse events following vaccination. In collaboration with the regional veterinary services directorates and district veterinary personnel, the study will ensure that an adequate supply of vaccines and consumables are maintained at the district level to meet the anticipated demand for vaccination services. Additionally, each veterinary personnel will receive quarterly airtime vouchers worth GHC 50 to support their efforts in delivering vaccination services.

**Fig. 3:**
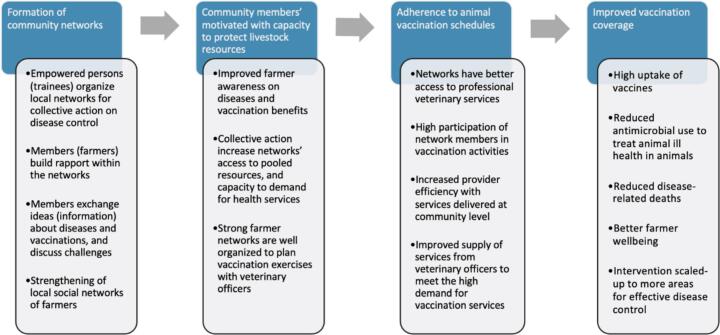
Illustration of the hypothesized theory of the intervention’s effect

A radio sensitization program will be held to inform all livestock farmers in both study arms about the vaccines availability. The program will be broadcast live on local radio stations in each study district, and the recordings repeated over two weeks prior to intervention implementation to sensitize the communities. The broadcasts will be done in the local languages by veterinary personnel in the study districts, with support from the research team. Fig. 3 below shows the theory of the intervention effect.

### 3.4 Theory of intervention effect

Please see Fig. 3 for details

### 3.5 Sample size

The sample size was estimated with the epiDisplay package in R software [28]. With a sample size of 460 farming households across 23 village clusters per treatment group, the study is powered to detect a difference of 25% to 40% vaccination coverage with 80% power, assuming alpha = 0.05, power = 0.8, intra-cluster correlation coefficient = 0.2, and non-response rate = 10%.

### 3.6 Data collection and management

Data will be collected at baseline, midpoint (6 months) and endpoint (12 months). At baseline, we will collect data on antimicrobial use on animals (frequency and quantity used per month). At midpoint, we will obtain data on vaccination uptake, antimicrobial use, and farmer wellbeing. In addition, we will review the registers maintained by the veterinary officers and village leaders, and conduct in-depth interviews with the stakeholders to evaluate intervention fidelity, acceptability, and feasibility to scale-up. Data collected at endpoint will include the data collected at midpoint in addition to data on disease-related mortalities experienced over the 12 months. The data will be collected with Open Data Kit (ODK) application, and interviews recorded.

All livestock-owning households in the study area who are geographically accessible all year round are eligible to be included in the study. To be included, farmers need to own at least three (3) tropical livestock units (TLUs) of eligible ruminants i.e. ruminants that are at least 3 months old. One (1) TLU corresponds to 0.75 cattle and/or 0.1 small ruminants (sheep and goats) [29]. Caretaker farmers who do not own the minimum TLUs of the livestock in the herds kept will be excluded from the study.

### 3.7 Data analysis plan

We will conduct an intention-to-treat analysis using the available case population, i.e. including data for all households with at least one follow-up observation. The analysis would adhere to principles outlined in ICH Statistical Principles for Clinical Trials [30]. Descriptive analysis will be done to assess for balance of key variables in study arms. If there are any imbalances in the key variables, the study team will discuss the findings, and post-hoc analysis conducted in sensitivity analysis.

### 3.8 The analysis plan for each study outcome (primary and secondary) is described below

#### 3.8.1 Primary outcome

We will compare the proportion of animals and herds of households vaccinated with CBPP and PPR vaccines between the study arms at mid- and- endpoints, relying on data from veterinary registers and the farmers’ follow-up surveys. We will compare the animal vaccination uptake at farm and district levels (average proportion of animals per herd, and average proportion of all herds per district vaccinated with CBPP, and PPR vaccines) between CAVI intervention households and controls, using mixed effect models with the logit function, accounting for villages as random effects.

#### 3.8.2 Secondary outcome (SO) 1

The study will compare between the study arms the average antimicrobial usage intensity (usage per animal per month) by farmers in treating animal diseases at mid- and- endpoints, using multilevel Poisson regression models, accounting for the hierarchical structure of the data. We will determine if changes in intensity of antimicrobials used from baseline to end of study is significantly different from zero.

#### 3.8.3 SO 2

In this analysis we will compare the percentage of disease-related mortalities in herds for each ruminant species reared, between CCAVI intervention and control households at the endpoint, using mixed effect models with the logit function, and accounting for the hierarchical structure of the data.

#### 3.8.4 SO 3

We will also compare the average wellbeing scores of the farmers between the study arms using linear mixed effect models, accounting for the hierarchical structure of the data. Wellbeing on the physical, psychological, social, and environmental domains will be assessed using the WHO Quality of Life BREF tool. The analysis will determine if the changes in farmers’ wellbeing scores from midpoint to end of study is significantly different from zero.

#### 3.8.5 SO 4

Registers of trainees and veterinary personnel, and the interview transcripts will be analyzed at mid- and- endpoints, for positive and negative experiences with CCAVI’s implementation, to determine acceptability of CCAVI. We will apply the framework proposed by Hermann et al. [31] to determine scalability.

We will also assess the cost per additional livestock vaccinated, as well as the program’s effectiveness in improving quality of life.

### 3.9 Quality control

The intervention has been registered on the Pan African Clinical Trials Registry, and the study protocol approved by the ethics review committee in Ghana (details provided below). The registers maintained by veterinary personnel will be reviewed quarterly to identify any shortfalls in service delivery, vaccine quantities, and consumables, allowing for appropriate remedial actions in collaboration with the Veterinary Services Directorate. Cold chain boxes will be provided to veterinary personnel and vaccine cooling refrigerators will be installed in each study district to maintain vaccine potency.

A monitoring and evaluation plan will be implemented to ensure the project achieves its intended design and objectives. Monitoring and evaluation activities will occur at quarterly intervals throughout the project lifecycle. Process evaluation will be conducted six months after the intervention’s implementation, involving a review of study registers, in-depth interviews with trained personnel, and observation of project activities to assess the acceptability, feasibility, and fidelity of the intervention protocol. In the second year, outcome evaluation will include a sero-surveillance survey to measure the impact of CAVI on livestock immunity levels. Monitoring and evaluation of vaccination coverage sustainability will also be conducted in collaboration with local veterinary authorities.

The key indicators for assessing intervention fidelity and outcomes will include the number of individuals trained, number of veterinary officers engaged, number of communities reached, vaccination coverage rates, and livestock health status. Throughout the monitoring and evaluation process, local stakeholders including veterinary officers, agricultural department personnel, and community leaders, will be actively involved to enhance the accuracy of data collection, foster collaboration, and support the integration of findings into future interventions. This approach aims to ensure sustainability and scalability of the intervention.

### 3.10 One Health implementation

This study has adopted a transdisciplinary approach, whereby an intervention proposed by non-scientific end-users (lay farmers) is evaluated by harnessing expertise from different scientific disciplines including veterinary medicine, public health, biostatistics, economics, microbiology and psychology. The study also takes into account the systematic relationship between policy and lay facets of the study context to enhance intervention success.

The relevant authorities at the national and local levels will be involved in the project implementation to enhance uptake of the results. The study will evaluate the acceptability and feasibility to scale of the intervention to foster sustainability of vaccination coverage achieved during this study. The study also makes use of the existing vaccination delivery infrastructure and provides tools that augment the service delivery to reinforce further intervention sustainability. The added value of this study will be an enhanced localized platform of farmers within each district that are able to exchange information on better animal health practices, harness the group strength to share vaccination costs and improve access to vaccination services, enhance better vaccination uptake and control of infectious diseases with its attendant benefits to farmers and the population.

### 3.11 Ethical considerations

The study was reviewed and approved by the Ghana Health Service Ethics Review Committee (GHS-ERC: 015/02/24). The trial is registered on the Pan African Clinical Trials Registry at: pactr.samrc.ac.za/ (No. PACTR202405854213937). Permission was obtained from the local authorities in the veterinary services directorates, and the districts. The purpose of the study, assurance of confidentiality and right of withdrawal will be explained to participants and informed consent sought. The consent form details all information about the research including background, purpose, potential risks and benefits. All records from this study will be treated as confidential records. Information collected on ODKs will be anonymized in any reports or publications from this study. Access to data collected will strictly be restricted to the principal investigator, and collaborators.

In recruiting research participants, no coercion will be used and no inducements will be offered. However, each participant will receive a token of airtime valued at GHC 50.00 over the three survey rounds after the questionnaire administration, to compensate for their time expended to partake in the study. The researchers declare no conflict of interest. To ensure fidelity to the intervention design, veterinary registers will be reviewed quarterly to identify shortfalls in veterinary personnel service delivery, vaccines, and consumables, for appropriate action in collaboration with the Veterinary Services Directorate. Cold chain boxes will be provided to veterinary personnel, and vaccine refrigerators provided for each district, to maintain vaccine potency.

## 4 Discussion

To the best of our knowledge, this is the first study to evaluate the effectiveness of a community-driven intervention aimed at addressing animal vaccination uptake challenges in sub-Saharan Africa. Recent global challenges with zoonotic disease control have underscored the need for strong inter-sectoral collaboration and community engagement in mitigating the impact of complex health challenges. The use of transdisciplinary approaches holds promise in fostering stakeholder ownership of public health interventions alongside improving their efficiency, effectiveness, and comprehensiveness [[32], [33], [34]]. Given that the main barriers to vaccination uptake in Ghana encompass both demand and supply factors, an initiative with extensive stakeholder involvement including livestock farmers, veterinary professionals, and local authorities stands to offer significant benefits to the disease control efforts. We have engaged the veterinary services at the national, regional and district levels in developing intervention protocols and training modules, ensuring that the materials are tailored to the context for achieving sustainability. However, such integrated end-user oriented initiatives have not been robustly evaluated, leaving a gap in evidence on strategies to enhance livestock vaccination uptake. There is thus a pressing need for a deeper understanding of how to incentivize end-user participation in vaccination program organization within rural settings.

Our intervention aligns with current global and regional strategies aimed at systematically and effectively tackling the burden of transboundary animal diseases, particularly in Africa, Asia and the Middle East in order to mitigate their adverse impact on food security and livelihoods of poor households. For example efforts have been directed towards the control and eradication of Peste des Petits Ruminants (PPR) by 2030 [35], alongside strategies implemented to tackle the burden of Contagious Bovine Pleuropneumonia (CBPP) [36]. By providing training and sensitization to farmers in our current study, we expect an enhanced uptake of livestock vaccination in Ghana, thereby reducing the occurrence of the targeted infectious diseases, alleviating the strain on the veterinary system, and minimizing the need and use of antimicrobials in livestock production. The anticipated improvement in livestock productivity is expected to contribute to better wellbeing of livestock dependent populations and enhance food security. Moreover, integrated in our intervention design is a process evaluation, which aims to evaluate the intervention’s fidelity, its acceptability to stakeholders, and the feasibility of scalability, with the aim of potential future expansion to other regions.

Our study has some limitations. Primarily, due to the nature of the intervention design, blinding is not feasible, potentially leading to non-blinding bias wherein dropouts could vary between the study arms. To mitigate this issue, both study arms would receive sensitization information on the availability and effectiveness of livestock vaccination for the target diseases (CBPP and PPR). Additionally, participating farmers will be provided with airtime vouchers to compensate for their time, similar to the vouchers provided to the trained individuals tasked with organizing the local platforms. There is also a possibility of non-compliance with the study protocols among the trained individuals, veterinary professionals and research assistants. To minimize this occurrence, the principal investigator has provided training for all research assistants on the study protocols and tools. Additionally, training has been provided to at least two farmers per intervention village and to veterinary professionals in the study area. Our training modules emphasize the importance of equitable representation and participation in decision-making, especially for women and young farmers. The intervention will be reinforced at the midpoint, and quarterly monitoring visits conducted in collaboration with regional and national veterinary service authorities are planned to identify instances of non-compliance and provide timely feedback. The study will document the lessons learned from project implementation through the process evaluation, thereby informing future delivery strategies.

## 5 Conclusion

We present the protocol of a cluster randomized controlled trial designed to evaluate the effectiveness of a community-led initiative aimed at improving the uptake of CBPP and PPR vaccines by livestock farmers in Ghana. The goal is to reduce the disease burden, the need for, and use of antimicrobials in livestock farming and minimize disease-induced livestock mortalities, thereby improving farmers’ wellbeing and food security in the region. The trial has been designed in collaboration with the end-users and local authorities to respond to the veterinary system challenges pertaining to livestock disease control. Should the intervention be effective, we aim to adapt and scale up CCAVI to other regions facing similar challenges.

## Consent for publication

Not applicable.

## Availability of data and materials

All data generated or analyzed during this study will be included in the published article [and its supplementary information files].

## Funding

This study is funded by Science for Africa Foundation to the Developing Excellence in Leadership, Training and Science in Africa (DELTAS Africa) programme [Afrique One-ASPIRE, Del-15-008 and Afrique One-REACH, Del-22-011] with support from Wellcome Trust and the UK Foreign, Commonwealth & Development Office and is part of the EDCPT2 programme supported by the European Union. For purposes of open access, the author has applied a CC BY public copyright license to any Author Accepted Manuscript version arising from this submission. The funders have no role in the study.

## CRediT authorship contribution statement

**Francis Sena Nuvey:** Writing – review & editing, Writing – original draft, Visualization, Project administration, Methodology, Investigation, Conceptualization. **Günther Fink:** Writing – review & editing, Validation, Supervision, Resources, Methodology, Conceptualization. **Jan Hattendorf:** Writing – review & editing, Visualization, Validation, Supervision, Resources, Methodology, Conceptualization. **Daniel T. Haydon:** Writing – review & editing, Validation, Supervision, Methodology, Conceptualization. **Gilbert Fokou:** Writing – review & editing, Validation, Supervision, Resources, Methodology, Conceptualization. **Kennedy Kwasi Addo:** Writing – review & editing, Validation, Supervision, Resources, Methodology, Conceptualization. **Jakob Zinsstag:** Writing – review & editing, Validation, Supervision, Resources, Methodology, Conceptualization. **Clemence Esse-Dibby:** Writing – review & editing, Validation, Supervision, Project administration, Methodology, Funding acquisition, Conceptualization. **Bassirou Bonfoh:** Writing – review & editing, Validation, Supervision, Resources, Project administration, Methodology, Funding acquisition, Conceptualization.

## Declaration of competing interest

The authors declare that they have no competing interests.

## Data Availability

Data will be made available on request.

## Appendix A Supplementary data

Supplementary material: Training Manual for the Creation and Operation of village-level Livestock Farmer’s Cooperative (Platforms). Image 1
