# Supplementary material for: Effects of community action on animal vaccination uptake, antimicrobial usage, and farmers’ wellbeing in Ghana: study protocol for a cluster-randomized controlled trial
Source: One Health. 2024 Dec 15;20:100952. doi: 10.1016/j.onehlt.2024.100952 (PMC11732147; doi:10.1016/j.onehlt.2024.100952)
Supplement: Supplementary file 1 — Supplementary material: Training Manual for the Creation and Operation of village-level Livestock Farmer’s Cooperative (Platforms). [file mmc1.pdf]

Training Manual for the Creation and Operation of village-level Livestock Farmer’s Cooperative (Platforms)

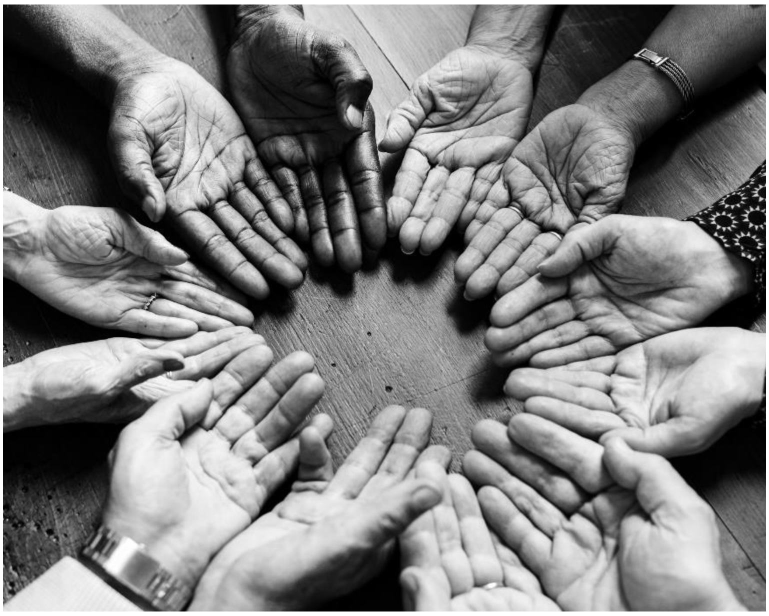

Afrique One REACH

Centre Suisse de Recherches Scientifiques en Côte d'ivoire

April 2024

Table of Contents

Introduction and Overview 3

Module 1 4

Session 1A: Building Good Relationships for Better Livelihood 4

Session 1B: Understanding Community and its role in Collective Action 5

Session 1C: Creating and Managing a Farmer Cooperative 6

Module 2 9

Session 2A: The burden and impact of CBPP and PPR on livestock productivity 9

Section 2B: Schedules for CBPP and PPR vaccines in Ghana 11

References 12

Introduction and Overview

This training manual was developed within the framework of the Afrique One Research Excellence for African Challenges in Health (REACH) Programme, which is funded through the Developing Excellence in Leadership Training and Science in Africa (DELTAS Africa II). DELTAS Africa is a long-term programme of the SFA Foundation supported with funding from Wellcome, and the Foreign Commonwealth and Development Office (FCDO) in the UK and administered by Centre Suisse de Recherches Scientifiques en Côte d'Ivoire. This manual has adapted the training guidance on creating cooperatives provided in two manuals to suit the current goal (Agriculture and Agri-food Canada, 2005; ILO, 2018). The goal of this training manual is to support the development of village-level livestock farmers' platforms in Ghana.

Within the agricultural sector, farmers' associations are pivotal entities that serve diverse functions, which could be broadly classified into two categories. Firstly, farmers' associations engage in economic and commercial activities, such as marketing, input supply, technical assistance, extension services, credit provision, information dissemination, and risk management. Secondly, these associations sometimes commit to advancing the collective interests of the farming community or specific segments of society. They cater to a defined membership or group of farmers, delivering tailored benefits to their constituents. The focus of this training manual lies in nurturing the establishment of farmers' association that advance the interests of livestock owners particularly in terms of effective control of priority diseases.

The training manual is to be used by the research team working with a group of livestock farmers who reside in villages allocated to the intervention arm of the study titled "The effects of collective community action on the uptake of animal vaccination services, antimicrobial usage, and farmers' wellbeing in Ghana". The training manual provides a structured approach for trainees to establish cooperative platforms within their local communities to overcome barriers to vaccination access for their livestock. Trainees will collaborate with veterinary officials to plan vaccination activities. The manual consists of two modules, designed to be completed sequentially. Module 1 focuses on the essential steps required to establish and operationalize a farmer association, emphasizing a paradigm shift in mindset and practice. It encourages farmers to grasp the potential of collective action to effect meaningful change in their lives. Central to this is cultivating an understanding of cooperative principles and the role of farmer associations in fostering community resilience. Farmers' associations will only thrive when members are actively engaged and perceive tangible benefits from collaboration. It outlines a developmental process encompassing awareness, understanding, commitment, and action, essential for the successful establishment and operation of farmer associations.

In our engagements with Ghanaian livestock farmers and veterinary service providers, the farmers and veterinary personnel demonstrated a good awareness and understanding of the negative impact of animal diseases on the productivity of livestock and have expressed their willingness to contribute to efforts that address the disease burden sustainably (Nuvey et al., 2023a; Nuvey et al., 2023b). In Module 2, the manual details the principles and effectiveness of livestock vaccines, as well as the personal and community level benefits, and the schedules for different vaccines for priority livestock diseases. Research had identified vaccination as the most effective strategy for controlling most infectious livestock diseases in sub-Saharan Africa with good returns on investment (Nuvey et al., 2022). Studies had also identified contagious bovine pleuropneumonia (CBPP) and foot and mouth disease (FMD) in cattle and Pestes de petits ruminants (PPR) and mange disease as priority diseases affecting the livestock sector in Ghana (Nuvey et al., 2023c). Among these diseases prioritized by the key stakeholders, CBPP and PPR have existing vaccines that are underutilized; only 15% of farmers regularly vaccinating their herds (Diop et al., 2011; Nuvey et al., 2023a).

The training package lasts approximately 2 – 3 hours, and is to be delivered after the baseline survey of the research project, and is reinforced at the midpoint survey.

Module 1

In this module, the farmers are introduced to the importance of collective action and the essential steps for establishing and managing a farmer cooperative association.

Session 1A: Building Good Relationships for Better Livelihood

In this session, the farmers engage in activities designed to foster mutual understanding and cooperation. The emphasis is on building strong relationships and recognizing the value of collaboration with others. Furthermore, the guide underscores the importance of cultivating positive relationships in the communities farmers live in, and among their peers. Cooperation enables farmers to leverage each other's strengths and perspectives for mutual benefit. These relationships serve as vital resources for achieving common objectives. Relationships thrive when there is reciprocal contributions and shared goal(s) by all parties.

Activity

In this exercise, each participant compile a list of all individuals who contribute to their livestock rearing efforts *(5 minutes)*. Next, the participants select the three (3) most significant contributors (persons) from their list *(5 minutes)*. Following this individual reflection, participants share their lists and engage in a discussion on the importance of supportive relationships *(10 minutes)*. Participants are encouraged to reflect on how these relationships can lead to the discovery of new collaboration avenues and the realization of mutual benefits.

Session 1B: Understanding Community and its role in Collective Action

Community serves as the foundation for effective collective action within a cooperative setting. It is essential for members of a cooperative to learn about and understand each other. The synergy among cooperative members is crucial to establishing and sustaining cooperative endeavors. Collective action entails collaborative efforts by a group of individuals united in pursuit of a shared goal to improve their collective standing. This session aims to enable participants to recognize opportunities for collective action and discern situations where group efforts yield greater benefits than individual endeavors.

Activity

The goal of this exercise is to encourage participants to reflect on the significance of community in establishing a farmer cooperative or platform. The exercise will also stimulate critical thinking and foster an understanding of the practical applications of collective action within the context of community-driven initiatives. Participants will explore the community's potential to address various challenges encountered in livestock rearing. Additionally, they will identify the factors that may either facilitate or impede a cooperative's objectives (*10 minutes*). Divide participants into groups of 3 – 4 persons for focused discussions. Following this, they will collectively engage in analyzing the story of Kweku, a livestock farmer in Salifukrom. Through this narrative, participants will identify the constraints Kweku faces and brainstorm how collective action within his village could potentially enhance his livelihood (*15 minutes*).

Kweku has been rearing cattle and goats for about 5 years now. He started with 5 cattle and 10 goats and was hopeful of a growth in his herd size. However, every year a particular disease spread among his animals and some of them die. Even those that survive lose weight and it takes many months for them to regain their weight. Kweku decided to sell one of the animals to take care of the rest, but the prices offered were low in the market due to the leanness of the animal. Kweku heard about a vaccine that can prevent the diseases spreading among his herd from an uncle who is also rearing animals in Agogo. However, when he called the veterinary officer to express his interest in vaccinating his animals, the officer informed him that the vaccine need to be given to 50 animals when opened by the end of the day. Kweku now has to try to find other farmers in his village who are also willing to vaccinate their animals at the same time so that he will not pay for vaccine vial alone. The price for the vaccination also includes the fuel costs for the veterinary officer to come and administer it to the animals.

One day, his assemblyman, who is also a livestock farmer, invited Kweku and other famers in the village to attend a meeting. During the meeting, the farmers all realized that the diseases affecting their animals are similar. The assemblyman proposed to form a cooperative platform in the village, and is looking for other farmers to join them so that they can pool their resources

together and coordinate efforts for vaccinations in the future with the veterinary officer. The assemblyman informed them that he has learned that if about two-thirds of all the animals in the village get vaccinated against the disease, it will provide protection for all the other animals. This is because the animals who can fall ill to the disease, and spread it in the village reduces.

With their combined numbers, they are able to plan ahead of each vaccination period, meet the minimum requirement of the number of animals needed to use a vaccine vial, and share the transportation cost for the veterinary officer so that the individual farmer vaccination costs is cheaper. They also share knowledge and experiences about actions that enhance better productivity of their livestock and challenges they faced so they can learn from each other. Because of this, the farmers achieved economies of scale in vaccination, reducing individual costs and ensuring herd-wide protection. Furthermore, shared insights and best practices improved overall livestock productivity, leading to healthier animals with higher market value. Through collaboration and solidarity, Kweku and his fellow farmers transformed individual challenges into collective opportunities, safeguarding their livelihoods and building a sustainable future for their community.

Discussion points: Why is co-ordination important in this scenario? What things would encourage collaboration among the members of a farmers' group? (Look for answers such as trust, communication, repeated interaction among the members). What other situations can the participants identify where co-ordination is required for group action to provide benefits? Review the role of trust and communication in achieving co-ordination and collaboration.

Session 1C: Creating and Managing a Farmer Cooperative

In this session, we delve into the key aspects of farmers' associations and co-operatives, highlighting their diverse functions and invaluable contributions to agricultural communities. Farmers' associations come in various forms, each tailored to address specific needs and challenges encountered by farmers. For example, farmers' associations serve as vehicles for delivering essential services and fostering collective empowerment. They may offer technical expertise, operate as commercial enterprises supplying agricultural inputs like fertilizer and feed, extend credit facilities to members, or engage in research and development activities. Some associations prioritize non-commercial roles, focusing on advancing members' collective interests through unified action. Cooperatives, in particular, exemplify the spirit of collaboration and mutual support. Members join forces to tackle shared challenges or seize collective opportunities that they cannot address on their own. While profit generation may not be their primary goal, cooperatives aim to enhance members' economic, social, and cultural wellbeing. Guided by principles of honesty, openness, social responsibility, and compassion,

cooperative members work towards common goals, driven by a shared commitment to each other's success.

As we embark on the formation of community-level cooperatives/ platforms, it is crucial to uphold certain principles that will shape their operation and ensure success. These guiding principles include:

a) **Voluntary and Open Membership**: The cooperative should welcome all individuals in the community willing to accept the responsibilities of membership, without discrimination or exclusion.
b) **Democratic Member Control**: Every member has a voice in the decision-making process, ensuring that the cooperative operates in a democratic and participatory manner. It is important that the unique needs and inputs of both women and men farmers are taken into account in the cooperative/ platform decision making.
c) **Member Economic Participation**: Members are both owners and users of the cooperative's services, actively contributing to and benefiting equitably from its operations.
d) **Education, Training, and Information**: Continuous learning and knowledge exchange are essential for empowering members and fostering a culture of mutual support and development.
e) **Concern for Community**: The cooperative should prioritize the sustainable development of livestock productivity, and operate with a commitment to community wellbeing, addressing local needs and fostering social cohesion.

In addition to these guiding principles, the formation of cooperatives is most likely to be beneficial under the following conditions:

a) Problems and constraints cannot be solved individually. The problem can be better solved by collaborating with people who face the same problem.
b) Assistance needed to address the problem is not readily available from family, government, or other institutions. Cooperatives offer a promising avenue for accessing the necessary resources and support.
c) The advantages of membership (access to services, inputs, financial services, and information) outweigh the duties of membership (contribution of resources such as money, time, and skills).

Activity

The goal of this exercise is for participants to engage in deliberations focusing on the objectives, membership criteria, decision-making processes, and conflict resolution mechanisms within the cooperative platform (*30 minutes*). To start the session, participants

are encouraged to reflect individually on the advantages and disadvantages of forming or joining the platform, as well as the anticipated benefits they expect to derive (*5 minutes*).

Aim and Objective

Participants will deliberate on the objectives of the cooperative platform within the context of this project. This includes fostering information and knowledge exchange on disease control measures, specifically vaccination, and promoting collective efforts in scheduling vaccination activities for eligible livestock among members. Additionally, participants will explore perspectives on how funds for planned vaccination activities should be managed, whether through central collection by designated leaders or individual members' responsibility. Discussion points will cover when members should allocate funds before a vaccination cycle. It may be important to challenge the participants' assumptions about the objective of the cooperative/ platform, and for what purposes the funds of members could be used (*8 minutes*).

Membership

All individuals residing within the designated intervention communities, who are willing to abide by the responsibilities of membership, are eligible to join the cooperative. The discussion will focus on strategies to promote and enhance the participation of women and young farmers in the cooperative. Additionally, participants will deliberate on whether membership should be limited to one member per livestock farming household and if household representatives are permissible within the platform. Furthermore, the group will explore whether non-participation in vaccination activities disqualifies an individual from membership. The participants should discuss the obligations of each platform member based on the project concept. Participants deliberate whether all members should have equal rights and responsibilities or if there should be different classes of members, such as the trained representatives or leaders tasked with specific roles like outreach, record keeping, and liaising with veterinary services. Additionally, the discussion will touch upon whether non-members can benefit from platform activities and the level of participation expected from each member. The process for joining the platform/ cooperative will be examined, including the criteria for approving interested individuals and the approval process itself. Lastly, participants will explore the circumstances under which membership may be terminated, and the associated conditions (*10 minutes*).

Decision-making processes

During this phase, it is essential to navigate methodically through the various on the decision-making processes envisaged for the cooperative/ platform, ensuring that participants grasp the nuances of each decision point.

Begin by exploring how major decisions will be made, including the voting or decision rules to be employed. Participants should gain clarity on the mechanisms for reaching consensus or making determinations within the association/ platform. Next, delve into the duties of the association/ platform leader, outlining their responsibilities and role in facilitating effective governance and coordination. In the event of a conflict or disagreement within the platform, discuss the available options for resolution, considering mechanisms such as mediation, or consensus-building. Participants should also contemplate the potential consequences for the platform if the interests of certain members are prioritized over others. Highlight the importance of equitable representation and participation in decision-making processes to ensure fair and inclusive governance. Conclude by briefly underscoring the need for equal representation and participation across all demographic groups in decision-making processes, fostering a sense of ownership and inclusivity within the association/platform (*7 minutes*).

Take a 15 minutes Health Break

Module 2

In this module, the participants delve into the negative impact of CBPP and PPR on the livestock sector, and the principles and effectiveness of livestock vaccines for CBPP and PPR, as well as the personal and community level benefits, and the schedules of the vaccines.

Session 2A: The burden and impact of CBPP and PPR on livestock productivity

Contagious bovine pleuropneumonia (CBPP) and Pestes des petits ruminants (PPR) are transboundary animal diseases with significant, and negative impact on livestock productivity and food security in many countries in sub-Saharan Africa including Ghana. In most countries in the region, both diseases have been prioritized for effective control to minimize their impact. This session should last for about *20 minutes*, including the questions and answers session at the end of the information dissemination on the two priority disease conditions.

Burden and impact of CBPP on livestock productivity

CBPP also known as lung sickness in cattle is caused by a bacteria (*Mycoplasma mycoides subsp. mycoides Mmm*), which causes a considerable burden for cattle owners in many parts of Africa (FAO, 2013). The disease manifests gradually, persisting within herds and causing significant morbidity and mortality rates. In endemic areas with high prevalence, chronic infections are common, leading to poor body condition and low productivity over several months. Moreover, CBPP is a barrier to trade in live animals, adversely affecting meat exports.

Despite ongoing control efforts such as mass vaccination campaigns and stringent movement restrictions, there has been limited progress due to high costs, and growing public resistance. Although official guidelines discourage antibiotic treatment, its widespread use persists among farmers who invest substantial resources in procuring these antibiotics for the control of CBPP. The indirect costs of control and reduction in the value of livestock because of the presence of CBPP are not well documented but are believed to be significant (Mariner et al., 2019).

For effective disease control, it is imperative that clinical protection or cure contributes to reducing disease transmission (Mariner et al., 2019). Although elimination of infected animals is often the most effective way of reducing transmission, testing and slaughter can be cost-prohibitive without compensation for affected farmers. Vaccination thus remains an avenue for reducing susceptibility of animals, offering proven effectiveness and good returns on investment (Nuvey et al., 2022). Vaccines are drugs given to offer protection for animals against specific diseases before the target diseases occur. However, to effectively eliminate diseases, a significant portion of the livestock population (at least 70%) must be vaccinated to interrupt transmission. The CBPP vaccine needs to be taken at least once every year, for it to be effective. The sustainable delivery of control interventions requires shared responsibility among government, private sector, and communities.

Research has shown that infectious diseases cause approximately one in ten adult cattle and two in ten calves mortalities annually (Pradère, 2014). Previous studies have identified the main challenges to vaccination uptake in Ghana as both demand and supply driven. On the demand side, barriers primarily stem from farmers' limited awareness and misconceptions about the benefits of vaccines, and the financial burden associated with vaccine affordability. Particularly, the requirement for farmers to bear the full cost of vaccine vials, even if they do not own a sufficient number of animals to utilize an entire vial. Supply-side barriers are mainly due to the limited number of professional veterinary officers accessible to farmers, and inadequate veterinary health infrastructure, which restricts availability and accessibility of veterinary services when required (Nuvey et al., 2023a).

Strengthened partnerships among all stakeholders are essential to improving vaccination uptake. Leveraging resources from farmer cooperatives with support from the public sector can address financial constraints in CBPP control and contribute to combating antimicrobial resistance by reducing disease prevalence and antibiotic use.

Burden and impact of PPR on livestock productivity

Peste des petits ruminants (PPR) is a highly contagious viral disease caused by the small ruminant Morbillivirus, and primarily affects sheep and goats (OIE, 2018). The disease manifests acutely and is characterized by fever, eye and nasal discharges, diarrhea and

pneumonia, and lesions on different mucous membranes particularly in the mouth. Once introduced, the virus can infect up to 90% of a flock, resulting in mortality rates ranging from 30 and 70% among infected animals (about 5 out of every 10 goats/sheep infected). The annual direct impact of PPR is estimated at USD 1.2 to 1.7 billion (OIE and FAO, 2015).

Since 2014, a global strategy for PPR control and eradication by 2030 has been established, with a focus on strengthening veterinary services, enhancing diagnostic capabilities for surveillance, and improving vaccination coverage. Effective implementation of this strategy is expected to significantly reduce the negative impact of PPR worldwide (OIE and FAO, 2015). In Ghana, PPR has been identified as a priority disease due to its detrimental effects on sheep and goat productivity (Nuvey et al., 2020; Nuvey et al., 2023c).

Research demonstrates the high efficacy of PPR vaccines, offering prolonged immunity to vaccinated herds. Vaccination also delivers positive returns on investment for households and supports public food security and improved nutrition (Nuvey et al., 2022). Despite prioritization of PPR and the availability of effective vaccines, vaccination uptake remains critically low, hampering efforts to reduce herd susceptibility. PPR elimination is most likely to be achieved if all goats/ sheep in an area receive the vaccine (but at least 8 out of every 10 goats/ sheep) Thus, concerted efforts are needed to mobilize collective action among all stakeholders in the livestock sector to address existing challenges and enhance vaccination utilization for better disease control.

Section 2B: Schedules for CBPP and PPR vaccines in Ghana

Access to CBPP and PPR vaccines in Ghana is facilitated exclusively by professional veterinary personnel. Farmers residing in rural areas where livestock is frequently reared need to coordinate with public veterinary officers within their respective districts to schedule vaccination appointments. Currently, vaccines are primarily stocked at the national and regional directorates of veterinary services. As part of this project, our objective is to enhance vaccine accessibility by establishing the necessary cold chain infrastructure at the district level.

Both PPR and CBPP vaccination typically occurs before the rainy season, mainly between May and June, when livestock herds congregate for feeding and watering. Animals eligible for vaccination include all cattle, sheep, and goats aged at least 3 months. Additionally, in southern Ghana, a secondary rainy season occurs between September and November, offering another opportunity for vaccine administration before this period.

Participants are encouraged to consider these recommended vaccination timelines during their local platform/cooperative deliberations to optimize the benefits of vaccination efforts. The session should last for about *20 minutes*, including a question and answer session.

References

Agriculture and Agri-food Canada, 2005. China Canada Agriculture Development Program Farmers' Association Development Strategy and Training Program. Centre for the Study of Co-operatives.

Diop, B., Daborn, C., Schneider, H., 2011. PVS Gap Analysis Report - Ghana. OIE, Paris, France, https://www.woah.org/app/uploads/2021/03/pvsgapanalysis-report-ghana.pdf.

FAO, 2013. Global Framework for the progressive control of Transboundary Animal Diseases. GF-TADs for Africa-Minutes SC8 8th Steering Committee Accra, Ghana June 2013. FAO, Rome, 30p, https://openknowledge.fao.org/handle/20.500.14283/bl419e.

ILO, 2018. Think.COOP: An orientation on the Cooperative Business Model. International Labour Organization (ILO) Bangkok, https://www.ilo.org/publications/thinkcoop-orientation-cooperative-business-model.

Mariner, J., Elidrissi, A., Raizman, E., 2019. Control of contagious bovine pleuropneumonia – A policy for coordinated actions. FAO Animal Production and Health Paper no 180. FAO, Rome, 52pp, https://openknowledge.fao.org/handle/20.500.14283/ca3949en.

Nuvey, F.S., Arkoazi, J., Hattendorf, J., Mensah, G.I., Addo, K.K., Fink, G., Zinsstag, J., Bonfoh, B., 2022. Effectiveness and profitability of preventive veterinary interventions in controlling infectious diseases of ruminant livestock in sub-Saharan Africa: a scoping review. BMC Veterinary Research 18, 332, https://doi.org/10.1186/s12917-022-03428-9.

Nuvey, F.S., Fink, G., Hattendorf, J., Mensah, G.I., Addo, K.K., Bonfoh, B., Zinsstag, J., 2023a. Access to vaccination services for priority ruminant livestock diseases in Ghana: Barriers and determinants of service utilization by farmers. Preventive Veterinary Medicine 215, https://doi.org/10.1016/j.prevetmed.2023.105919.

Nuvey, F.S., Hanley, N., Simpson, K., Haydon, D.T., Hattendorf, J., Mensah, G.I., Addo, K.K., Bonfoh, B., Zinsstag, J., Fink, G., 2023b. Farmers' valuation and willingness to pay for vaccines to protect livestock resources against priority infectious diseases in Ghana. Preventive Veterinary Medicine 219, 106028, https://doi.org/10.1016/j.prevetmed.2023.106028.

Nuvey, F.S., Kreppel, K., Nortey, P.A., Addo-Lartey, A., Sarfo, B., Fokou, G., Ameme, D.K., Kenu, E., Sackey, S., Addo, K.K., Afari, E., Chibanda, D., Bonfoh, B., 2020. Poor mental health of livestock farmers in Africa: a mixed methods case study from Ghana. BMC Public Health 20, 825, https://doi.org/10.1186/s12889-020-08949-2.

Nuvey, F.S., Mensah, G.I., Zinsstag, J., Hattendorf, J., Fink, G., Bonfoh, B., Addo, K.K., 2023c. Management of diseases in a ruminant livestock production system: a participatory appraisal of the performance of veterinary services delivery, and utilization in Ghana. BMC Veterinary Research 19, 237, https://doi.org/10.1186/s12917-023-03793-z.

OIE, 2018. Peste des petits ruminants (infection with small ruminant morbillivirus). Manual of Diagnostic Tests and Vaccines for Terrestrial Animals, 2018. OIE, Paris, France, https://www.woah.org/fileadmin/Home/eng/Health_standards/tahm/3.08.09_PPR.pdf.

OIE, FAO, 2015. Global strategy for the control and eradication of PPR. OIE, Paris, France, https://www.woah.org/app/uploads/2021/03/ppr-global-strategy-2015-03-28.pdf.

Pradère, J.P., 2014. Improving animal health and livestock productivity to reduce poverty. Rev. sci. tech. Off. int. Epiz. 33, 735-744, 723-734, https://doi.org/10.20506/rst.33.3.2315.
